# Evaluation of Informed Consent Practices for Off-Label Use of Anti-Dementia Medications in Patients with Vascular Dementia

**DOI:** 10.1192/bjo.2026.11446

**Published:** 2026-06-30

**Authors:** Nudrat Nudrat, Kalpa Wijesinghe

**Affiliations:** Merseycare NHS Foundation Trust, St. Helens, United Kingdom

## Abstract

**Aims:**

Vascular dementia (VD) is a progressive neurocognitive disorder associated with cognitive decline, behavioural and psychological symptoms, and reduced quality of life. No medications are licensed in the UK specifically for VD, yet cholinesterase inhibitors and memantine–licensed for Alzheimer’s disease–are frequently prescribed off-label based on clinical judgement. Off-label prescribing carries additional responsibilities under GMC, NICE, and UK Government guidance, including ensuring patients receive sufficient information about risks, benefits, and the unlicensed nature of treatment. Locally, Trust policy (MMP39) and the Pan Mersey Shared Care Agreement require explicit documentation of informed consent before prescribing responsibility is transferred to primary care. Within LLAMS St Helens, documentation of consent for off-label prescribing has been inconsistent, and capacity assessments have not always been decision-specific. This project aimed to evaluate current practice and improve the recording of informed consent, capacity, and best-interests processes for patients with VD prescribed antidementia medication.

**Methods:**

A retrospective review of clinical records was conducted for all patients diagnosed with VD and prescribed antidementia medication up to 15 March 2025. Data were extracted from RiO consultation notes, MDT records, and prescription summaries, focusing on documentation of consent, capacity, and risk/benefit discussions. Findings were presented to the LLAMS team, followed by an educational workshop reinforcing best practice and the need for explicit off-label consent. A prospective reaudit was completed from March to November 2025 to assess the impact of the intervention.

**Results:**

Cycle 1 (Pre-intervention): Forty patients were reviewed; 30 were prescribed antidementia medication. Of these, 6 (20%) had documentation confirming they were informed about off-label use, 18 (57%) had capacity recorded, and 25 (83%) had a documented risk/benefit discussion.

Cycle 2 (Post-intervention): Forty-one patients were reviewed, of whom 10 were started on antidementia medication. Six of these patients commenced treatment after the educational intervention. Among this group, capacity was documented in all cases (100%), with each recorded as lacking capacity. Risk and benefit discussions with next of kin were documented for 5 patients (83%), and 5 patients (83%) were informed about the off-label or unlicensed nature of treatment.

Compared with Cycle 1, documentation of off-label consent improved from 20% to 83%, and decision-specific capacity recording increased from 57% to 100%, indicating a positive shift in practice following the intervention, although documentation remained variable overall.

**Conclusion:**

This project demonstrates that while risk/benefit discussions are generally well recorded, explicit documentation of off-label consent and decision-specific capacity assessments remains inconsistent. Early improvements following education suggest that structured prompts, enhanced MDT communication, and strengthened GP correspondence may support sustained change. Embedding a consent checklist and ongoing reauditing will help ensure transparent, patient-centred, and legally robust prescribing within LLAMS.

